# Blended e-learning and end of life care in nursing homes: a small-scale mixed-methods case study

**DOI:** 10.1186/1472-684X-13-31

**Published:** 2014-06-16

**Authors:** Conor JT Farrington

**Affiliations:** 1 Cambridge Centre for Health Services Research (CCHSR), Cambridge Institute of Public Health, Forvie Site, University of Cambridge School of Clinical Medicine, Box 113 Cambridge Biomedical Campus, Cambridge CB2 0SR, UK

**Keywords:** Training, E-learning, End of life care, Nursing home care

## Abstract

**Background:**

A ‘blended’ (e-learning and facilitated workshops) training course for Group C staff (i.e. staff with relatively infrequent contact with end of life care) has been delivered across several English counties with the aim of improving end of life care in nursing and residential care homes. This paper evaluates the impact of the course on participants’ understandings of and confidence in delivering end of life care in one nursing home, while also considering barriers to change in practice.

**Methods:**

A mixed-methods case study approach, incorporating pre- and post-course questionnaires (*SHA East of England End of Life Care Education Programme ‘ABC’ Project Work Force C or Non Nurse Workforce B Pre and Post Course Questionnaire; E-Learning in End of Life Care Study Pre and Post Course Questionnaire*), documentary analysis, semi-structured interviews, and observation of course workshops. Participants were 20 members of staff at a nursing home in a city in the East of England, including 14 Health Care Assistants (carers) and 6 others (administrative, activities, hosting, and catering staff). The questionnaires and interviews assessed understandings of and confidence towards end of life care delivery.

**Results:**

Improvements in participants’ confidence in delivering end of life care were observed, particularly in the core competency areas of symptom management, communication, and advance care planning. A shift towards more detailed and more holistic understandings of end of life care was in evidence; some participants also championed end of life care in the home as a result of the course. Several barriers to changes in practice were encountered, including uneven participation, the absence of mechanisms for disseminating new insights and knowledge within the home, and a widespread perception that nurses’ professional dominance in the home made sustainable change difficult to enact.

**Conclusions:**

While blended e-learning courses have the potential to generate positive change in participants’ understandings of and confidence about End of Life Care, organizational and inter-professional obstacles must be overcome in order to translate these changes into improved end of life care delivery in nursing (and residential) homes.

## Background

As Western populations age, increasing numbers of elderly people reside in nursing and residential care homes. In the UK, over 410,000 people now live in care homes (at least 350,000 of whom are in England and Wales alone), and around 20% of the UK population dies in care homes - a figure which rises to 36% in those aged 85 and over, and which is predicted to rise significantly in the future [1,2]. Elderly care home residents present multiple clinical challenges including multiple and progressive morbidity, cognitive impairment, general frailty, and psychosocial distress [3,4]. While residents may not be at imminent risk of death upon entering their care home, they are nevertheless highly likely to die there. Consequently, end of life care, broadly defined as an approach that ‘treats, comforts, and supports individuals who are living with, or dying from, progressive or chronic life-threatening conditions,’ [5] is an integral and essential part of the care provided by care homes and by those occupations working within care homes, including ‘healthcare assistants’ (carers), nurses, activities staff, administrative staff, and catering staff. The provision of high-quality end of life care in nursing and residential care homes has been shown to reduce emergency admissions to hospitals, reduce levels of distress in residents, families and care home staff, improve staff-resident communication and adherence to residents’ wishes, and, more widely, promote greater openness about death and dying among staff [1-3,5,6]. However, barriers to the provision of such care include variable levels of training, high staff turnover, underfunding and excessive staff workload, misunderstandings about end of life care, emotional and spiritual challenges, and wider societal taboos relating to talking about death and dying [1,7-10].

A range of clinical and policy initiatives have emerged over the past decade in response to these and other challenges in end of life care delivery, including the Department of Health’s 2008 *End of Life Care Strategy*[1,3,8,10,11]. This document emphasised the need to ensure that all staff involved in end of life care possess the requisite knowledge, skills, and attitudes through training strategies such as e-learning. Indeed, e-learning is described as ‘essential’ in the Strategy, in light of the numbers of staff involved and the diversity of their training needs [12]. Nationally, a dedicated e-learning programme offers over 150 e-learning sessions on end of life care to ‘Group B’ staff (i.e. practitioners who frequently provide end of life care as part of their role, such as Accident & Emergency staff). In one English region, a local e-learning programme (referred to as the ‘ABC’ course; see below for details) has adopted a ‘blended’ approach - i.e. face-to-face facilitated workshops alongside online content - to deliver end of life care training for ‘Group C’ staff (practitioners such as carers in nursing homes, who provide end of life care less frequently than Group B staff).

Critics of e-learning raise concerns such as varied levels of internet accessibility, digital literacy, and participant motivation [12,13]. By contrast, e-learning advocates highlight a number of benefits, including (for organisations) reduced training costs and ease of monitoring workforce skill levels, and (for individuals) flexibility, ease of accessibility, and self-driven learning [12-14]. For many observers, these benefits are particularly associated with ‘blended’ e-learning programmes incorporating a mix of ‘classroom’ and distance-based learning, witnh the latter often facilitated by internet access and, increasingly, the use of smartphones and tablets) [12-14]. More generally, it is known that end of life training can improve knowledge, skills, and attitudes, but researchers have yet to evaluate the impact of blended e-learning training programmes such as the ABC course on end of life care in nursing and residential care homes [15,16]. Consequently, the extent to which blended e-learning courses enable care home staff to acquire appropriate knowledge, skills, attitudes, and confidence for the delivery of high-quality end of life care remains opaque. This paper addresses this gap by evaluating the impact of a particular blended e-learning course - the aforementioned ABC course - on members of staff (including, but not exclusively, carers) in one nursing home in the East of England, focusing in particular on (a) staff members’ understandings of and confidence in delivering end of life care and (b) barriers to translating new understandings into practice. The study was guided by the following research questions: Can a blended e-learning training programme generate positive change in participants’ understandings of, and confidence in delivering, end of life care in care homes? And what are the main barriers to translating new understandings into practice?

## Methods

### Research design

This study adopts a mixed-methods case study approach. Case study methodology can be defined as the in-depth exploration of ‘a program, an event, an activity, a process, or one or more individuals’ in which the case or cases are ‘bounded by time and activity, and researchers collect detailed information using a variety of data collection procedures over a sustained period of time’ [17]. In this study, data collection methods included self-completion questionnaires, semi-structured interviews, and participant observation; as such the study exemplifies a mixed-methods approach, defined by Cresswell as a ‘methodology for conducting research that involves collecting, analyzing, and integrating (or mixing) quantitative and qualitative research (and data) in a single study or a longitudinal program of inquiry… [on the basis that] qualitative and quantitative research, in combination, provide a better understanding of a research problem or issue than either research approach alone’ [17]. This approach allows for triangulation (corroboration from different methods) and complementarity (elaboration and clarification of results from different methods), thus adding depth and insight to analysis.

### Research setting

The research setting for this study was Elm House (a pseudonym), a medium-sized nursing care home with a total of 68 staff members, including 16 nurses, 31 healthcare assistants (carers), and 21 other administrative, maintenance, activities, domestic, and catering staff. Elm House is owned and managed by a national private provider of health and social care, and is registered to provide accommodation for up to 60 residents who require nursing or personal care, including those with dementia and mental health needs.

The study focused on the delivery and impact of the aforementioned ABC course at Elm House from June-October 2012, with additional data gathering from January to March 2013. The ABC course was commissioned by a regional health body, whose subject matter experts (in both educational and clinical fields) worked in partnership with a private learning provider to develop the learning materials on the basis of the aforementioned national end of life e-learning programme. The overarching intention of the ABC course is to render these materials more accessible to carers and other Group C staff members within nursing and residential homes. The online component of the course consists of six modules each taking around an hour to complete, with an introductory session on overarching principles of end of life care followed by four modules focusing on each of the four core end of life care competencies as developed in the national end of life Strategy – communication, comfort and wellbeing, assessment and care planning, and advance care planning – and a final module outlining tools for end of life care (including the Liverpool Care Pathway, Gold Standards Framework for Care Homes, and the Preferred Priorities of Care document). Each module consists of three sections: *Learn*, in which participants learn required skills and knowledge through text, images, animations, and questions; *Listen*, in which participants listen to and reflect upon case studies between residents and carers; and *Practise*, with opportunities to engage with model situations. There are also several offline activities for each module. The e-learning interface was designed to be attractive and user-friendly, and incorporates features such as a help button, a glossary of key terms, the capacity to personalize the interface (e.g. regarding text size and font colour), an audio narrator function to support those who have difficulty reading English, and a telephone support line for those experiencing IT difficulties.

The blended component of the course consists of several facilitated workshops for course participants, with an introductory session incorporating an emotive DVD about end of life care followed by five workshops held to clarify, discuss, and reflect upon, in a retrospective and practice-focused manner, the material presented in the first five online modules, and subsequently a final workshop to discuss end of life tools and also participants’ action plans for the future. These meetings are facilitated by nurses with significant experience in end of life care delivery and training, who also made themselves available to participants for additional contact via email both during and after the ABC course. In Elm House, the workshops were facilitated by two experienced nurses, and took place in the home’s sitting rooms. Each participant was awarded ten hours’ backfilled time for these workshops, meaning that they were required to complete the e-learning component of the course in their own time.

### Study participants

In terms of inclusion/exclusion criteria for the study, potential participants were included if they had signed up to participate in the ABC course, and excluded if they had not signed up to participate in the ABC course. A total of 20 staff members opted to participate in the ABC course at Elm House, thus generating a pool of 20 potential study participants from a range of occupational backgrounds and demographic characteristics (Table 1). However, only 60% (N = 12) of these staff members had finished the course at the end of data collection (Table 2). This drop-out rate was described by the course facilitators as unusually high; reasons given by participants in post-course interviews included lack of time, perceptions of irrelevance, personal reasons (including sickness and family bereavements), and the lack of internet facilities at Elm House. For related reasons, difficulties were also encountered in terms of data collection, with a number of participants failing to complete questionnaires administered both by the ABC educational facilitators and the author, such that only 55% (N = 11) participants completed both the pre- and post-course SHA questionnaire and only 30% (N = 6) participants completed both the pre- and post-course author-designed freetext questionnaire (Table 2). Furthermore, staff departures from Elm House and staff concerns about time made it impossible to interview more than 75% (N = 15) of participants. Consequently, the numbers of study participants varied unavoidably between different aspects of data collection. However, all participants who completed both pre- and post-course questionnaires were also interviewed, thus ensuring the greatest possible congruity between different aspects of data collection.

**Table 1:** ABC participants at elm house by occupational background and demographic characteristics

**Occupational background**	**Age group**				**Gender**		**English as first language**		**Total**
	**18-29**	**30-41**	**42-53**	**54-65**	**F**	**M**	**Y**	**N**	
Health Care Assistants (Carers)	7	3	2	2	9	5	8	6	14
Administrative staff	1	0	0	0	1	0	1	0	1
Activities staff	0	1	1	0	2	0	2	0	2
Catering staff	1	1	0	0	0	2	2	0	2
Hosting staff	0	0	1	0	1	0	1	0	1
Total	9	5	4	2	13	7	14	6	20

**Table 2:** ABC participants at elm house by occupational background and participation in ABC course and data collection

**Occupational background**	**Nos. of participants**	**Completed course**	**SHA questionnaire completed**			**Author questionnaire completed**			**Interviews**
			**Pre**	**Post**	**Both**	**Pre**	**Post**	**Both**	
Health Care Assistants (Carers)	14	8	14	8	8	9	6	4	10
Administrative staff	1	1	1	1	1	1	1	1	1
Activities staff	2	1	1	0	0	1	0	0	1
Catering staff	2	1	2	1	1	1	1	0	2
Hosting staff	1	1	1	1	1	1	1	1	1
Total	20	12	19	11	11	13	9	6	15

### Data collection

Data collection focused on participants’ perceptions of end of life care and their own practice rather than clinical outcomes *per se*. The audit of deceased resident notes (see below) represents a partial exception in that this part of data collection can be understood as indirectly measuring the impact of the ABC course on end of life care outcomes via ‘process’ measures (i.e. procedures carried out to and for patients while providing care [5]) such as advance planning discussions.

Data collection was conducted using a range of quantitative and qualitative methods, including: a 36-item NHS SHA East of England pre- and post-course self-completion questionnaire for course participants (see below; Additional file 1); an 8-item author-designed freetext pre- and post-course self-completion questionnaire for course participants (Additional file 2); documentary collection (specifically a pre/post course audit of clinical notes for deceased residents, detailed in Additional file 3); author-conducted semi-structured interviews with course participants (N = 15, as mentioned above) and the care home manager (total N = 16); and author-conducted observations of seven facilitated workshops at Elm House.

The SHA questionnaire (Additional file 1) was developed, piloted and revised by NHS SHA East of England (the former regional health body) with the objective of evaluating changes in the confidence of individual Group C staff members with regard to delivering end of life care in the following four EoLCS core competencies: assessment and care planning (12 items); symptom management and well-being (6 items); communication (12 items); and advance care planning/end of life tools (6 items) [11]. The questionnaire incorporates a four-point Likert scale, with 1 = not at all confident and 4 = very confident. The questionnaire was administered by ABC course facilitators as part of standard course evaluation. While the questionnaire possesses face and content validity, the regional health body piloted and revised the questionnaire without regard to the instrument’s psychometric characteristics (i.e. construct validity and internal reliability, with the latter measured by Cronbach’s alpha); as such data generated from this questionnaire must be interpreted with some caution.

The author-developed freetext questionnaire (Additional file 2) was developed by the author with reference to key themes in literature on end of life care (6 items) and e-learning (2 items), and tested for face validity with the ABC course facilitators and piloted with research colleagues before being administered to study participants. The questionnaire was developed and administered in order to augment the data generated by SHA questionnaires with more detailed and individual responses in terms of participants’ changing knowledge of and understandings of important facets of end of life care (including the aims, timing, tasks, responsibilities involved in end of life care) and e-learning (including online resources and potential contribution to training needs). Freetext or open-ended questions were chosen in order to allow respondents to answer in their own terms and/or offer unusual responses, with fewer constraints placed on possible answers when compared with closed questions (as in the SHA questionnaire) [18]. Owing to the small sample size (N = 6) for completed pre- and post-course questionnaire, reliability testing was not carried out; neither was validity testing, which in any case is challenging to determine for open-ended questions, which typically generate qualitative rather than quantitative data [19].

With regard to documentary collection, patient records were assessed by ABC course facilitators as part of their standard evaluation procedure, utilizing a 21 item pre/post course audit of anonymized clinical notes for 10 deceased residents of Elm House (5 pre-course and 5 post-course), with regard to three categories of care: advance care planning (11 items); anticipatory planning (7 items); and communication/coordination (3 items; see Additional file 3).

In terms of semi-structured interviews, participants were recruited on a purposive sampling basis in order to obtain as wide a range of professional backgrounds as possible in proportion to the total numbers of participants from each group (Table 2). The care home manager was also interviewed, but is not included in Table 2 as the manager did not participate in the ABC course. Interviews were semi-structured and retrospective, conducted according to an interview topic guide developed (as with the freetext questionnaire) in light of literature on end of life care and e-learning, and focusing on four general areas: defining end of life care; end of life care training; the ABC course; and enablers of/barriers to long-term change (see Additional file 4). Interviews were digitally recorded and took place in Elm House, lasting between 20 and 40 minutes. Informed verbal consent, incorporating a discussion of the purpose of the study, research and analytical methods, anonymity, and eventual dissemination, was obtained from all participants before interviews took place. Written consent was not sought, on the grounds that seeking formal written consent in contexts such as Elm House (where research rarely takes place) could unnecessarily heighten concerns regarding the research process and potential eventual use of data, thus potentially undermining participants’ trust in the author as a researcher and negatively influencing researcher-participant interactions in interview settings and elsewhere.

The seven facilitated workshops observed spanned the breadth of the ABC course and included the initial and final workshops in addition to five workshops focused on different aspects of the course (see above). Workshops took place in one of two meeting rooms in Elm House, which were reserved for the purpose so that residents, residents’ families, and Elm House staff who were not participating in the course were not present. Workshops took place at a number of different times in order to allow staff members working on various different rotas to participate; as a consequence, there were considerably fewer participants at each workshop observed than the overall number of course participants. The average number of participants in the seven observed workshops was 3.9. The workshops took the format of facilitated discussion led by the ABC course facilitators with reference to online materials, and lasted between 60 and 90 minutes. The author recorded the content of discussions in the workshops using handwritten notes rather than a digital recorder owing to perceived discomfort with the recorder on the part of some participants.

### Data analysis

The research design described above generated both quantitative and qualitative data. The quantitative data generated by administering the SHA questionnaire and the pre/post course audit of deceased resident notes were analysed using descriptive statistics to produce measures of central tendency (i.e. arithmetic mean) and dispersion (i.e. standard deviation). The small sample sizes involved precluded more detailed statistical investigations that would have been possible with sample sizes greater than N = 30.

Interviews were digitally recorded with interviewees’ consent, and subsequently transcribed and analysed using an iterative ‘thematic analysis’ approach [20] and Atlas.ti software. Handwritten transcripts of workshop observations and freetext data from the author-designed pre/post course questionnaire were then analysed in light of the thematic framework developed through thematic analysis. Thematic analysis is designed to allow themes to emerge from the data in an iterative manner through six sequential analytical steps: familiarisation with the data; generating initial codes; searching for themes; reviewing themes; defining and naming themes; and producing a final analysis [20]. The trustworthiness and authenticity of the resulting analysis was addressed by consulting the ABC course facilitators; this approach was taken to avoid placing further time burdens on participant by asking them to fulfil additional participant validation functions [18].

### Research ethics

The ethical status of the research was established using the appropriate online resources (i.e. the NHS Health Research Authority Decision Tool) provided by the UK National Research Ethics Service, which confirmed that the study was service evaluation and so did not require formal ethical approval. Other approvals and permissions were gained from relevant bodies, and all individual participants were asked for their permission for the data to be collected and used as part of the evaluation. All data were kept securely on a password protected computer and all hard copy data was kept in a locked room within a secure filing cabinet. For the purpose of this study specific care home data and participants’ responses have been anonymised.

## Results

The findings are presented in two principal sections, which, in line with the study’s mixed-methods approach, [17] present key findings thematically rather than with primary regard to the specific quantitative or qualitative data collection methods employed (although distinctions are made for the sake of clarity between the various methods utilized). In line with the overarching research questions, the two broad themes presented below relate, respectively, to: (a) changes in confidence and understandings regarding end of life care; and (b) barriers to change in practice. The first section demonstrates that the ABC course has led to demonstrable improvements in participants’ understandings of, and confidence in delivering, end of life care, whereas the second section discusses briefly some of the organizational challenges that were encountered in terms of translating participants’ learning into practice at Elm House.

### Changes in understandings and confidence regarding end of life care

Focusing on changes in ABC course participants’ understanding and confidence regarding end of life care, this section canvasses quantitative findings arising from the NHS SHA questionnaire and the audit of deceased resident notes alongside qualitative findings arising from the author-designed freetext questionnaire, semi-structured interviews, and workshop observations.

#### NHS SHA questionnaire

**Figure 1:**
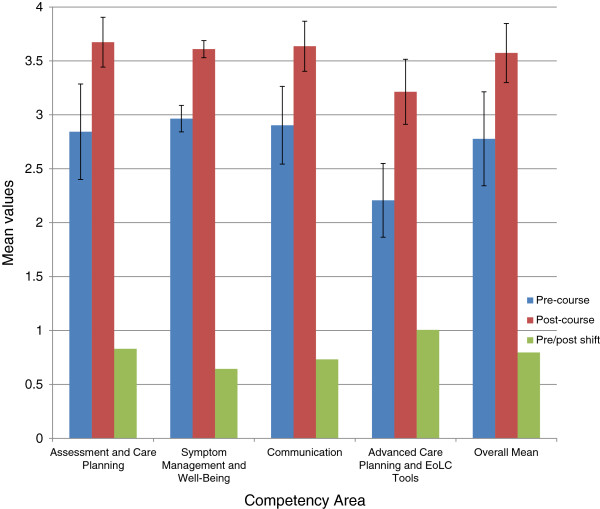
Pre-post course shifts in ABC participants’ levels of confidence in core competency areas (SHA Questionnaire).

The principal findings from the NHS SHA-designed questionnaire are presented in Figure 1, which indicates pre-post course shifts in mean levels of participants’ confidence in the four EoLCS end of life competency areas - assessment and care planning, symptom management and well-being, communication, and advance care planning/end of life tools [11] - and an overall average across all areas (weighted by number of items), for the 11 participants who completed both pre and post course questionnaires. Figure 1 also shows the standard deviations observed in each competency area.As demonstrated in Figure 1, the largest increases in mean levels of confidence were evident in the areas of assessment and care planning (average pre-post shift of 0.83 on a four point scale, or 29.2%) and advance care planning/end of life care tools (average pre-post shift of 1.0, or 45.6. The advance care planning/end of life care tools area also demonstrated three of the six highest pre-post shifts for individual questionnaire items. However, this competency area also showed the lowest post-course level of the four end of life competency areas (3.21 compared to an average of 3.58) and the five lowest post-course mean values for individual items, suggesting a potential need for further training in this area.

The areas of communication (average pre-post shift of 0.73, or 25.2%) and symptom management and well being (average pre-post shift of 0.64, or 21.2%) also showed strong improvement, in addition to above-average post-course confidence levels (3.64 and 3.6 respectively, compared to an overall post-course average mean confidence level of 3.58). The six items with the lowest increases in pre-post confidence levels were all at or above the overall average post-course confidence levels, thus showing little room for further improvement.

The overall average improvement in mean confidence levels was 0.8, representing a 28.7% advance in confidence across all competency areas.

#### Resident notes audit

**Figure 2:**
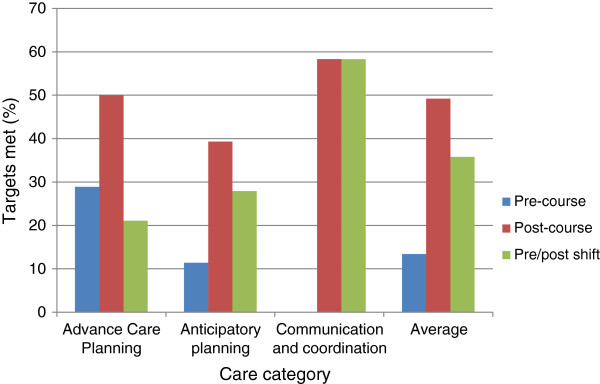
Pre- and post-course audit of deceased patient records at elm house.

Figure 2 presents the findings of the audit of anonymised patient records of 10 deceased residents (5 pre and 5 post course) at Elm House. Patient records were assessed by ABC course facilitators with regard to the extent to which they met targets derived from end of life care best practice within three care categories: advance care planning; anticipatory planning; and communication/coordination (Additional file 3). The patient records of one of the deceased residents in the post-course audit were missing owing to an administrative oversight at Elm House, and it was unclear from four of the pre-course notes and two of post-course notes whether or not residents died in their preferred place of death; the pre/post comparison has been weighted accordingly. All three care categories assessed showed noteworthy improvements in terms of meeting care targets, with improvements in specific categories as follows: 21.1% (advanced care planning), 27.9% (anticipatory planning) and 58.3% (communication/coordination), demonstrating an average overall shift of 35.8%. The dramatic improvement in the area of communication is noteworthy insofar as it contrasts with the more uniform improvements demonstrated in mean confidence levels across all four end of life care competency areas, including communication. Since (as previously mentioned) the resident notes audit indirectly measures end of life care outcomes, this finding suggests that communication may be the competency area in which the most immediate impacts of end of life care training may be expected.

A notable exception to the general trend of improvement in terms of meeting end of life care targets relates to the prescription and dispensing of syringe drivers and the use of the Liverpool Care Pathway, which were 0% in both pre and post audits, thus highlighting specific needs for further training in these areas.

#### Freetext questionnaire

Proceeding now to consider qualitative findings, the principal findings from the author-designed freetext questionnaire are presented in Table 3, which documents shifts in understandings of end of life care for the 6 participants (2 non-carers and 4 carers) who completed both pre and post questionnaires. As Table 3 indicates, shifts were evident in a number of areas, including definitions of end of life care, which became more holistic, less defined by terminal illness, and more focused on ensuring the best possible death for residents. Thus one carer wrote in response to Item 2 (‘Main aims of end of life care’) that ‘EoL [end of life care] is a vital tool to give meaningful life to a person,’ while another expanded their pre-course definition of end of life care as keeping residents ‘comfortable’ to a post-course definition of keeping them both ‘comfortable’ and ‘happy’ (Table 3). There was also evidence of an increased understanding that all members of care home staff are responsible for (different aspects of) end of life care, with one carer noting in response to Item 4 (‘Responsibility for end of life care’) that whether care is delivered in a home or a hospital, it is delivered by a ‘collective team…[with] responsibility to carry out EoL [end of life] care.’

**Table 3:** Pre-post course shifts in participants’ understandings of/attitudes towards end of life care (author questionnaire)

**Item and content**	**Non-carer**	**Non-carer**	**Carer**	**Carer**	**Carer**	**Carer**
**1. Appropriate time to initiate end of life care**	Shift from physical to more holistic definition of appropriate timing for initiation of end of life care	Pre-course questionnaire left blank; post questionnaire defined end of life as the last year of life and/or initiated at any time, according to preferences	Shift from generic awareness of need for EoL care when ‘condition starts to deterioriate’ to a more precise ‘within the last year to six months of their life’	Shift towards more precise definition of timing for EoL care (from ‘upon admission’ to ‘1 yr prior to expected death’)	Shift from generic ‘upon admission’ statement to patient-focused concern – end of life care initiated when required and on advice of medical team	Shift towards broader understanding of timing for initiating end of life care (i.e. at point of admission rather than with diagnosis of impending death)
**2. Main aims of end of life care**	Shift from idea of keeping residents physically and emotionally comfortable to wider understanding incorporating ideas of dignity and respect	Pre-course questionnaire left blank; post questionnaire set out aims as ensuring residents’ needs are met and holistically treated	Slightly fuller definition of the aims of EoL care, from keeping residents ‘comfortable’ to keeping them ‘comfortable and happy’	Shift from minimal definition of EoL care as carrying out residents’ and families’ wishes to ensuring ‘that the person can have the best death possible’ and ‘to die with dignity and die how they want to’	Pre-course questionnaire gave fuller definition of end of life care aims, perhaps reflecting this carer’s previous nursing training	Shift towards more content in idea of end of life care, from ‘best care and understanding’ to ‘dignity, comfort, and meaningful life’
**3. Tasks and tools involved in end of life care**	Little shift; non-carer already recognised the wide range of tasks that can be involved in EoL care	Pre-course questionnaire left blank; post questionnaire showed awareness of pain relief, wellbeing, social inclusion, LCP, GSF, PPC	Mention in the post-course questionnaire of one of the EoL tools (LCP)	Left blank in pre-course questionnaire; in post-course questionnaire, substantial content including talking to patients, assessment, planning, implementation and evaluation of care, including aftercare	Both left blank	Fuller content in the pre-course questionnaire; post course merely states ‘EoL[end of life] is a vital tool to give meaningful life to a person’
**4. Responsibility for end of life care**	Post-questionnaire blank; pre-questionnaire recognises that everybody has a role in EoL care	Pre-course questionnaire left blank; post questionnaire stated that end of life care is everyone’s responsibility, but care staff in particular	Slight shift towards wider definition of who is responsible for EoL, from nurses, carers and doctors to nurses, carers, GPs, and families	Shift from staff looking after residents and family, to wider understanding of responsibility – ‘whatever organisation/home/hospital… it is a collective team…[with] responsibility to carry out EoL care’	Pre-questionnaire blank; post-questionnaire acknowledges that all staff involved with resident have responsibility for end of life care	No shift; carer already recognised the need for all occupations to be involved
**5. National guidelines and policies**	Post-questionnaire blank; ‘Not sure’ in pre	Pre-course questionnaire left blank; post questionnaire mentioned the Mental Health Act	Both left blank	Significant shift from ‘government [web]sites’ to mention of Gold Standards Framework, Advanced Care Planning, Preferred Priorities of Care, Mental Capacity Act, powers of attorney, deprivation of liberty	Pre-questionnaire blank; post-questionnaire notes Gold Standards Framework and the Liverpool Care Pathway	Shift towards recognition of importance of dementia and vulnerable adults guidelines, and relevance of end of life to a wide range of policy areas
**6. Necessity for specialist training in end of life care**	Post-questionnaire blank; pre-questionnaire recognises training as important	Pre-course questionnaire left blank; post questionnaire stated that training was ‘very necessary… to understand our residents preferences to ensure they have the best possible care’	No shift: both pre and post stated that end of life care training was ‘useful’, without elaborating	No shift in recognition of importance of course (‘very important’) but carer added ‘I have learnt so much from this course’	Pre-questionnaire blank; post-questionnaire recognises the need for specialist training in end of life care, as ‘it is the last thing you can do for somebody before they die’	Little shift; carer had already recognised the importance of training
**7. Sources of information on end of life care**	Post-questionnaire blank; pre recognises internet, in-house training, policies	Pre-course questionnaire left blank; post questionnaire mentioned books, leaflets, and video	Both left blank	Shift towards wider awareness of resources, from ‘internet, books etc’ to ‘videos, books, government websites, internet, colleagues, policies’	Both left blank	Slight shift from internet resources to ‘all sources of info’
**8. Contribution of e-learning to end of life training**	Post-questionnaire blank; pre-questionnaire emphasises the need for time to carry out e-learning	Pre-course questionnaire left blank; post questionnaire mentioned e-learning as a source of information	Both left blank	Little substantive shift; pre questionnaire stated that e-learning can make a ‘big contribution’ while the post questionnaire stated that e-learning allows users to ‘be educated and learn about certain topics’	Both left blank	Shift from generic remark on the importance of training towards recognition of e-learning in particular – ‘easy and convenient’

In substantive terms there was also some increase in awareness of end of life care tools such as the LCP (Liverpool Care Pathway) and GSF (Gold Standards Framework) – an interesting finding in light of the absence of LCP awareness in the resident notes audit - and a slight increase in awareness of relevant policy instruments and resources, with the latter including relevant government websites, books, and colleagues. There was also some recognition of the value of e-learning, with one carer stating for instance in their post-course response to Item 8 (‘Contribution of e-learning to end of life care’) that e-learning allows users to ‘be educated and learn about certain topics.’

#### Interviews

In common with data generated by the SHA and freetext questionnaires and resident notes audit, interview findings revealed noteworthy, if varied, transformations in understandings among participants, especially with regard to attitudes towards end of life care and death and dying more widely. Participants expressed positive opinions regarding the course’s impact on their understandings of end of life care, which became both more detailed and more holistic.

In terms of detail, participants noted their increased knowledge of clinical aspects of end of life care, such as relevant care tools and terminal symptomatology:

It has helped me a lot in dealing a resident towards their end stage. In a way I learned things unfamiliar such as the … components of [the] LCP [Liverpool Care Pathway]. Also a deeper understanding of different symptoms that affect comfort and well-being.

Carer

I took on board from this [course] that people do down and they might come up a little bit … It’s not always a smooth fade out… Whereas I think prior to doing this [course] I just believed, “Well, they’re just going to die.”

Carer

In this context, one carer specified several specific ways in which the course had helped her to care for dying patients. Noting first that she no longer assumed that dying patients ‘don’t have to be in [the local hospital]’, she added that she had learned from the course that

you shouldn’t… put suction into somebody’s mouth when they’re a bit clogged up… I didn’t know that and certainly when I first worked here we had someone dying and the person in charge was using the suction machine… [But] you don’t have to do this and you can adequately clean somebody’s mouth with a soft toothbrush… [and] gently get that out… [It’s] little things you can do to make them more comfortable.

In terms of more holistic understandings of end of life care, one carer remarked on the difference between their pre-course, essentially administrative understanding of end of life care – ‘it was really just getting to a point when you knew someone was going to die’ – to a more expansive definition of end of life care involving ongoing and sensitive communication with residents and families in order to achieve a ‘good death’, now understood as helping residents’ families as well as residents themselves:

…[T]hrough doing this course I’m now very aware… I spoke to [one resident’s] daughter about their wishes and I said “I don’t want you to tell us now, I don’t want you tell us next week, go home, talk to your family about it and then bring it back to us when you’re ready,” …so I’d already instigated that [idea of] “What do you want for your dad when he dies?” … what we are doing is picking up on what we know they like, talking to the family about what they know about this person.

Another carer made similar remarks, summing up and emphasizing the importance of end of life care training given that ‘you only get one shot at getting someone’s death right.’

In addition to demonstrating more detailed and holistic understandings of end of life care after the course, most interviewees also demonstrated increased willingness and motivation post-course to discuss and implement end of life care in their own practice, including non-carer staff members who (pre-course) did not typically see themselves as having a role to play in delivering end of life care. Thus one non-carer remarked:

I now think about talking to [residents] about the end of life instead of just brushing it away… from this [course] it was like, well, maybe there is something that I can do… before I get a carer [to come and help].

Interestingly, this interviewee then added that one of the most important aspects of the course had been the way in which it confirmed and legitimised practices that were already taking place: ‘A lot of it I was already doing… it was good to have the confirmation that I was going the right way about things.’

Carers also noted that the course had given them confidence to address key issues involved in delivering end of life care:

I feel confident to discuss dying with patients and relatives. Before I wasn't sure or confident about discussing death and perhaps a little afraid of death myself. Looking after a dying person has become clearer and I now feel more comfortable with this.

I’ve certainly put [the training] into practice because we have had a few people die since… I took it upon myself to say right, we need to know their mouth care has been done… that they’ve been changed and they’re comfortable.

Several participants stated moreover that the course had given them confidence not just to deliver end of life care but also to address issues of death and dying in their own lives, and with regard to their own relatives and indeed themselves; thus one carer remarked that ‘[the] EoL [ABC course] made me confident, [and] have knowledge and Ideas. I can apply it not only in Home care but in my day to day life.’ This finding suggests that end of life care training of nursing home staff has the potential to aid recent governmental and charitable efforts to counter public reluctance to talk about issues of death and dying [11].

A small number of interviewees did attest to specific problems arising from the computer-based course component. Some of these arose from physiological challenges; one non-carer noted that ‘I can’t stand being on a computer for long because it really hurts my eyes’. Internet access was also a problem for one carer whose home computer was broken; when asked if it there wasn’t a computer suitable for use at Elm House, the carer replied: ‘No, not with internet.’ This serves to highlight the lack of internet access provided for Elm House staff, which could present problems for staff members without access to internet facilities at home or elsewhere. More widely, several interviewees referred to the high drop-out rate from the course, citing as an explanation the motivational challenges arising from e-learning and its out-of-hours requirements:

People [i.e. fellow course participants]… spoke about… the fact that if I go home it’s my time… and I’m not going to be paid for that time…People feel like it’s part of their time is being taken by work.

Carer

For those who persevered with the course, however, few mentions were made of difficulties encountered with the e-learning components, and the online material itself was seen as informative and user-friendly. One carer remarked for instance that e-learning is ‘a great way [for] people to access information and further education… [It is] very accessible’, while another described the internet portal as ‘easy to use.’

#### Workshop observations

As mentioned previously, the e-learning part of the course was supplemented by blended workshop sessions facilitated by the ABC Course leaders. The facilitated workshops served two key purposes, being at the same time opportunities for the facilitators to ensure that participants had understood the material and opportunities for course participants to clarify specific clinical and care-related points arising from the online material. This dual purpose is demonstrated by the following discussion of DNR (Do Not Resuscitate forms) and GSF (Gold Standard Framework), which occurred during the fourth workshop observed:

Facilitator (F): What forms do you remember apart from the DNR [Do Not Rescuscitate]? [Mentions the Advanced Decision to Refuse Treatment form.]… Does anyone have these kinds of forms here? Have these forms been implemented?

Carer 1 (C1): I don’t know.

F: You can find out if they have such forms. The forms are useful when patients can’t speak for themselves… You must all know who’s for resuscitation and who’s not. Can anyone think of cases where the forms would be useful?… Do you have any feeding tubes here?

Carer 2 (C2): Not any more.

F: It’s difficult to know when to stop feeding them. But with a form – it’s clearer when they want it and when they don’t.

C1: Is it giving them the right to give up?

F: Well, what are the benefits of the form?

Carer 3 (C3): It’s an opportunity for them to give up and have a better quality of life?

F: Everything is a balance with quality of life. It’s about having control – not necessarily giving up. What are the benefits of advanced care planning? It stops crises from happening… Let’s review what you know about the GSF. Have you heard about it?

C1: Heard about it, yes, but no more.

F: […] It’s about standardising care in the last year of life.

C3: Is that with the different coloured stars?

F: Yes. And the seven Cs? [Explains GSF colour-coding and the ‘seven Cs’: communication; coordination; control of symptoms; continuity; continued learning; carer support; care in the dying phase] … It’s all about good communication.

Non-Carer 1 (NC1): Sometimes if someone dies at the weekend, I might not know until late on Monday.

Non-Carer 2 (NC2): Sometimes communication falls through. I could be making a birthday cake for someone who’s passed away. We need a system to notify everyone.

F: That’s part of aftercare in the GSFs – for nurses or anyone. Because the administrative staff need to know, for their interactions with GPs, relatives, and so on.

In this context, one carer noted in a post-course interview that ‘It was nice to be able to ask somebody [i.e. the workshop facilitator], “Did I do right or did I do wrong?”

Moreover, despite many individual interviewees acknowledging the value of individual online learning (see above), the emotive and potentially distressing nature of some of the material led participants to emphasise how much they valued the facilitated workshops in terms of discussing and reflecting upon some of the challenging issues and topics covered in online learning. In this context, one non-carer remarked, ‘It was good to come in and talk about it [end of life care]’, while a carer noted that being a carer was ‘quite an emotional job’ and that the workshops allowed participants to acknowledge the emotional aspects of their work: ‘It brought it all to life… When you’re working sometimes you forget about everything [residents] have been through and it just makes you realize all over again… It’s very emotional.’ As such, it was clear that participants recognized the value of the blended e-learning approach.

With regard to participants’ views regarding changes in their own learning and practice, similar themes emerged in the workshops as in interviews and freetext questionnaires. When asked in the final workshop to sum up their learning from the course, responses included: confirmation of existing practice; heightened confidence; and increased ability to deal with death and dying residents. On this latter point, one carer elaborated:

I was a little afraid of dying before coming on this course, but now I’ve got the confidence to see it as a normal part of life and that helping people die with dignity is very important.

Two of the more experienced carers made particularly enthusiastic remarks about the course in the final workshop, describing the course as ‘really very good’ and ‘invaluable’ respectively, and noting how the course had transformed their attitudes towards death and dying. They also emphasized the ways in which they had put their learning into practice since the course, for instance by employing advanced care planning and the LCP (Liverpool Care Pathway), by ensuring a comfortable environment for dying residents, and by paying attention to aftercare for bereaved relatives, and, more widely, acting as ‘champions’ for end of life care in Elm House. The importance of champions has been noted in the literature and was also emphasized by the Elm House manager: ‘There’s a lot of champions around the place [who] are keen to be ahead of the game… and it has massive benefits’ [21,22].

### Barriers to change in practice

In addition to the improvements in understandings of and confidence towards end of life care delivery canvassed in the preceding section, qualitative findings from interviews and workshops also revealed areas in which challenges were encountered in terms of translating learning into practice at Elm House. Two themes in particular emerged: the lack of post-course discussion at Elm House, and inter-professional barriers between carers and nurses. The following sections address these themes in turn.

#### Lack of post-course discussion at Elm House

Several interviewees noted the lack of a regular forum at Elm House at which they can share learning experiences and establish a shared approach to end of life care. In the absence of such a forum, high workloads mean that there is little time to discuss issues with fellow staff members, other than occasional opportunities arising between tasks and/or shifts: ‘it’s just a chat, when you can get one’ (carer); ‘We just… talk, like in our breaks, just two minutes’ (carer). Interviewees noted furthermore that there had been no communication or dissemination activities undertaken with regard to the specific content of the ABC course. Added to the lack of regular opportunities to reflect upon and share ideas and experiences, this suggests that the impact of the ABC course on end of life care delivery is likely to be limited to those who completed the course.

Even within this latter group, moreover, some participants experienced a decline in enthusiasm after the end of the course. For example, one of the more experienced carers noted in an interview taking place three weeks after the end of the course that:

When we did the course, we were very up for talking to people [about end of life care issues]… [But] we’ve had new residents recently, and do you know what, thinking about it just now, I haven’t spoken to them about it… [I’ve] forgotten to.

As such, the apparent absence of structured efforts to disseminate ABC course learning may have limited the course’s long-term impact upon end of life care, in line with literature on sustainable educational interventions [23].

However, the Elm House manager noted that a number of changes regarding end of life care had recently been introduced at the home, partly as a result of the ABC course; these included: a communication book in which carers can write notes for nurses and GPs; keyworkers for each resident to communicate with family members; GSF colour-coding for all residents; DNAR and PPC documents; and increased use of the LCP. As mentioned above, the manager was very positive about end of life champions, and had also sought to gain maximum exposure to end of life care by putting as many staff members as possible on the ABC course. As such, there is evidence of systemic change alongside concerns regarding a lack of structured dissemination.

#### Inter-professional barriers

Literature on multi-disciplinary teamwork emphasizes the potential challenges of inter-professional collaboration owing to distinctive occupational cultures [24,25]. Findings did point to a barrier of a kind between carers and non-carers (i.e. those in administrative, catering, and other roles), with one non-carer remarking that it was difficult to interact with carers because they spent most of their time with residents and other carers:

[M]ost of the time I just see them in passing, we might get a few minutes to stop and talk but not a lot and not really in-depth about anything… I don’t … really have a lot of contact with the carers, because obviously if they’re washing and dressing people, it’s all behind closed doors, so I .. don’t really know what they’re up to.

However, in nursing homes such as Elm House the principal professional divide is typically that between nurses and carers, with carers stating in interviews and workshops that nurses were reluctant to take account of carers’ insights regarding residents (with whom carers spend more time) or end of life care expertise acquired from the ABC course. This reluctance, interviewees suggested, stemmed partly from a widespread nursing emphasis on physical health rather than more holistic issues, and partly from the relative failure of Elm House nurses to attend (separate) end of life training themselves. Relating an episode in which a junior carer had felt unable to challenge a nurse’s (mis)diagnosis of a distressed resident, a different carer stated that

[the carers have] some fantastic training but we’re not allowed to put it into practice… I have heard on many occasions nurses say, I’m the trained staff, you do like I say… but [carers] can very easily see that there’s something wrong with somebody.

Likewise, in the final workshop a carer stated: ‘I’d like nurses to take more notice [of carers]. Just because I’m not qualified in nursing doesn’t mean I don’t know anything.’ Another carer then added: ‘I was brushed off by the nurses several times… I shouldn’t be brushed off [by them].’ Non-nursing staff are not only frustrated by experiences such as this, but are also at risk of becoming disillusioned and disinterested in applying their training and learning experiences to their practice. In this context, one carer remarked ‘I think here [i.e. at Elm House], because the nurses aren’t on board with it [i.e. new perspectives on end of life care gained from the ABC course], then the carers feel less inclined to take it on.’

As discussed below, this study did not seek the perspectives of nurses themselves – an absence that should be born in mind when interpreting the findings presented above. In this context, it is worth noting one non-carer’s statement (in an interview) that carers can be ‘just as resistant’ to change as nurses: ‘It’s “We’ve been doing it this way, this way’s… worked for years, why change.’ This interviewee then continued: ‘Somebody will go: we’ll do it this way, and maybe for a couple of weeks or something [they will], but then something else comes up and they stop.’ Consequently, it is possible that carers have exaggerated the extent to which the barriers they face in translating new ideas into practice arise from *inter*-professional tensions and differences rather than *intra*-professional issues such as motivation and sustained change. Nevertheless, it is clear that the professional divide between nurses and carers at Elm House may impact negatively on the incorporation of carer-driven changes in practice.

## Discussion

This study aimed to utilise a mixed-methods, small-scale case study approach to evaluate the impact of a blended e-learning end of life care course on participants’ knowledge of and confidence towards end of life care delivery in one nursing home in England and barriers to translating these changes into practice, in order to establish whether this educational intervention has led to positive change in line with previous research on end of life care training [15,16] and blended e-learning more generally [12-14]. Taken together, the quantitative and qualitative findings presented above add to existing research by indicating that the ABC blended e-learning course in end of life care did in fact improve participants’ understandings of, and confidence in delivering, end of life care in Elm House. However, findings also furnished evidence of barriers to sustained improvement.In terms of changes in knowledge and confidence, firstly, the SHA questionnaire indicated a noteworthy 28.7% post-course advance in mean levels of confidence across all four end of life care competency areas (assessment and care planning, symptom management and well-being, communication, and advance care planning/end of life tools; see Figure 1). A particularly strong advance (45.6%) was noted in the area of advance care planning/end of life tools. However, the audit of resident care notes demonstrated a lower level of post-course improvement in terms of meeting care targets in this area (21.1%) as opposed to two other areas (anticipatory planning, 27.9%; communication/coordination, 58.3%), and no demonstrable knowledge of an important end of life care tool (the Liverpool Care Pathway, or LPC), suggesting that increased participant confidence in advance care planning and end of life care tools may not translate well into improved outcomes. Conversely, the strong increase in communication/coordination targets met in the audit of deceased resident notes suggests that communication may be the end of life competency area in which training may lead to the most dramatic improvements.

In line with findings generated using quantitative methods, qualitative findings from the author-designed freetext questionnaire, semi-structured interviews and observed workshops also pointed towards substantial improvements in participants’ knowledge of and confidence towards end of life care. In particular, participants evidenced transformed understandings of end of life care, moving away from their previous, narrowly-defined conceptions of end of life care as ‘terminal’ care and towards new understandings that were at once more detailed (in clinical terms) and more holistic (in caring terms). Participants, that is, not only became substantially more aware of key medical aspects of terminal symptomatology and relevant care management tools, but also demonstrated an understanding of the need to involve residents and their relatives in dialogue and planning concerning end of life care. Staff members participating in the ABC course emphasized their increased confidence in discussing the many difficult issues surrounding death and dying both at work and elsewhere, expressed their appreciation of the opportunity to discuss emotive issues in the ABC facilitated workshops, and (in general) welcomed the e-learning aspects of the course. In conjunction with the findings generated using quantitative methods, these findings suggest that the ABC course has led to substantial improvements in course participants’ knowledge of and confidence towards end of life care, in addition to demonstrating the potential of training and education to widen and enrich understandings of death and dying (in line with government end of life policies [11]).

In many ways, the emotive character of issues raised in end of life care highlights the particular benefits that derive from blended e-learning courses, as opposed to either face-to-face only or e-learning only courses [12]. As suggested by recent research, e.g. research on stigmas surrounding mental illness, [26,27] the e-learning components of blended courses allows learners to explore challenging issues – such as issues surrounding death and dying - in private, without fear of stigma or judgement; yet at the same time, the face-to-face components of blended courses allows learners to discuss and explore issues with colleagues and facilitators, adding depth to understanding and allowing for the generation of shared viewpoints and mutual support [28]. This combination of privacy and collegial support is perhaps of particular importance for carers working in end of life care, given the frequent inter-professional tensions between nurses and carers and, at a more fundamental level, the substantial emotional and psychological burdens arising from carers’ day-to-day work and, in particular, the family-like bereavements experienced when familiar patients die [29].

In terms of barriers to sustained improvements in end of life care, secondly, qualitative findings revealed two key factors that may limit the long-term impact of the ABC course despite management-led systemic interventions to facilitate improved end of life care delivery. The first of these is the lack of structured, regular opportunities for reflection and discussion among staff members. While this is a common feature of pressurized work environments in which staff work long shifts, it does highlight the possibility that discussion of ABC course learning may be limited to course participants rather than serving as a focal point for change across the wider working community at Elm House. The second key barrier to sustained improvements is that presented by inter-professional tensions, particularly between carers and nurses. While carers often spend more time with residents than nurses owing to the nature of their roles, carers perceive nurses as disregarding carers’ perspectives and insights and prioritizing a less holistic, more physical health-oriented model of end of life care. In addition to further limiting the dissemination of ABC course learning, this perception also demotivates and demoralizes carers, limiting their enthusiasm to apply their changed understandings in practice.

Consequently, this small-scale case study supports the view that, while blended end of life training for carers can lead to important changes in understanding and confidence, training alone is insufficient to guarantee sustained impacts on care delivery [3,5]. Research suggests that critical factors for successful, long-term change include: communicating a clear vision about the need for change; gaining commitment and participation from all staff members; communicating during the change; and making the change a permanent feature of organisational culture [30,31]. In the context of nursing homes seeking to improve their end of life care, taking account of these factors would entail a joined-up approach to end of life training across the two key staff groups of nurses and carers in order to overcome inter-professional barriers, ensure communication and wider commitment to change through (e.g.) regular meetings and updates, and embed end of life care into organizational routines and systems.

It should be noted that this study exhibits a number of limitations. With the exception of the audit of deceased resident notes, the study focuses on participant perceptions and understandings rather than direct or indirect clinical outcomes. The generalisability of the study’s findings is relatively low, in common with most case studies, and is negatively impacted by relatively small numbers of participants and by the possibility that other nursing homes with different organizational frameworks may exhibit distinctive, potentially significantly different, results. As mentioned above, the study does not include the perspective of nurses at Elm House, nor does it present the findings of long-term evaluation taking place several months or years following the educational intervention. Lastly, the study does not separately examine the impact upon educational outcomes of different modes of educational delivery, such as e-learning only, workshops only, and blended e-learning (e-learning and workshops). Future research could usefully address these limitations by undertaking evaluation of end of life care educational interventions with larger numbers of study participants across multiple nursing homes with distinct organizational frameworks, by evaluating direct and indirect clinical outcomes as well as educational outcomes, by evaluating different modes of educational intervention, by seeking the perspectives of all staff members (including nurses), and by undertaking long-term follow-up evaluation.

## Conclusion

This mixed-methods small-scale case study demonstrates that blended e-learning courses have the potential to generate positive change in participants’ understandings of and confidence towards end of life care. Course participants evidenced more detailed and more holistic understandings of end of life care and greater confidence across a range of end of life core competencies. However, the study also revealed specific areas where further training is needed alongside wider organizational and inter-professional barriers to long-term sustainable change, highlighting the need to address organizational change alongside educational interventions.

## Competing interests

The author declares that they have no competing interest.

## Authors’ contributions

The author was solely responsible for designing, conducting, analyzing, interpreting, and writing up the study described above. All authors read and approved the final manuscript.

## Pre-publication history

The pre-publication history for this paper can be accessed here:

http://www.biomedcentral.com/1472-684X/13/31/prepub

## Supplementary Material

Additional file 1 SHA East of England end of life care education programme ‘ABC’ project work force C or non nurse workforce B pre and post course questionnaire. Click here for file

Additional file 2 E-learning in end of life care study pre and post course questionnaire. Click here for file

Additional file 3 Items in pre and post course deceased patient record audit. Click here for file

Additional file 4 Topic guide for interviews. Click here for file
