# Supplementary material for: Blended e-learning and end of life care in nursing homes: a small-scale mixed-methods case study
Source: BMC Palliat Care. 2014 Jun 16;13:31. doi: 10.1186/1472-684X-13-31 (PMC4080686; doi:10.1186/1472-684X-13-31)
Supplement: Additional file 1 — SHA East of England end of life care education programme ‘ABC’ project work force C or non nurse workforce B pre and post course questionnaire. [file 1472-684X-13-31-S1.doc]

**Blended e-learning improve end of life care in nursing homes:**

**A small-scale mixed-methods case study**

**Additional file 1: SHA East of England End of Life Care Education Programme ‘ABC’ Project Work Force C or Non Nurse Workforce B Pre and Post Course Questionnaire**

## East of England End of Life Care Education Programme ‘ABC’ Project Workforce Pre and Post Course Questionnaire

Participant Name:

Mentor Name:

Date Education Programme Commenced:

Date Education Programme Completed:

Please complete this questionnaire at the beginning and end of the programme.

Please rate each competency using the key below.

Confidence questionnaire key:

1 – Not at all confident

2 – A little confident

3 – Quite confident

4 – Very confident

| Competencies: **Assessment and Care Planning**  NHS KSF: HWB 2, 3, 4, 5, 6, 7, C3, C6 | | | Pre-course | Post-Course |
| --- | --- | --- | --- | --- |
| 1 – Not at all confident, 2 – A little confident, 3 – Quite confident, 4 – Very confident | | | | |
| A1 | Enables or undertakes appropriate essential personal care ( e.g. mouth care, skin care, positioning, managing elimination and personal hygiene, within agreed guidelines and protocols whilst supporting independence as far as possible | |  |  |
| A2 | Contributes holistically to the care of a dying patient with regard to pain and other symptoms. | |  |  |
| A3 | Demonstrates regard for the privacy, dignity and cultural diversity of both patient and informal carer and obtains valid consent prior to undertaking any procedure or care | |  |  |
| A4 | Recognises the physical, psychological, cultural, spiritual, social, financial, legal and ethical issues affecting patient and informal carers | |  |  |
| A5 | Involves informal carers in personal care and/or contact where appropriate | |  |  |
| A6 | Recognises and reports to senior staff members any distress of patients and informal carers | |  |  |
| A7 | Recognises the emotional, spiritual and social needs of patents and informal carers showing awareness of use of specialised (palliative care) and other resources | |  |  |
| A8 | Identifies and reports any significant perceived changes/deterioration in the patients condition | |  |  |
| A9 | Uses and accurately completes an appropriate integrated care pathway such as the LCP | |  |  |
| A10 | Undertakes last offices in accordance with clinical guidance and organisational policy | |  |  |
| A11 | Respects cultural diversity and religious beliefs and practices | |  |  |
| A12 | Recognises own limitations of knowledge or experience and refers to other colleagues in own or wider teams | |  |  |
| Competencies: **Symptom Control and Comfort and Well being** | | | Pre- Course | Post Course |
| 1 – Not at all confident, 2 – A little confident, 3 – Quite confident, 4 – Very confident | | | | |
| SM1 | | Contributes to the assessment and management of symptoms, resulting in appropriate care planning at the end of life |  |  |
| SM2 | | Identifies commonly experienced symptoms in End of Life Care:  Constipation |  |  |
| SM3 | | Agitation |  |  |
| SM4 | | Pain |  |  |
| SM5 | | Breathlessness |  |  |
| SM6 | | Nausea and Vomiting |  |  |

| Competencies: **Communication** NHS KSF: C1; IK1 | | | | Pre Course | Post Course | |
| --- | --- | --- | --- | --- | --- | --- |
| 1 – Not at all confident, 2 – A little confident, 3 – Quite confident, 4 – Very confident | | | | | | |
| C1 | | Listens for, recognises, acknowledges and explores cues from dying patients and informal carers wanting to talk about concerns, preferences and wishes | |  |  | |
| C2 | | Demonstrates a range of appropriate communication skills including:  Empathic listening Use of silence  Reflection Open and closed questions | |  |  | |
| C3 | | Recognises and responds accordingly to fear, anger, denial and collusion particularly when this is impacting upon effective communication | |  |  | |
| C4 | | Acknowledges and recognises advance care planning wishes of patients and facilitates appropriately the conversations with relevant staff | |  |  | |
| C5 | | Acknowledges, recognises and responds with sensitivity and compassion to the needs of patients and informal carers irrespective of their backgrounds | |  |  | |
| C6 | | Provides general information confidentially to patients and carers but reports more complex information needs ( e.g.; details of patients condition or prognosis to staff member in charge) | |  |  | |
| C7 | | Records care delivered including significant interactions with patients and families according to local policy and procedure | |  |  | |
| C8 | | Effectively communicates information verbally to other members of the team using available handover opportunities | |  |  | |
| C9 | | Contributes to the presentation of the patient’s condition and emotional health of the patient and informal carer in a multi-disciplinary team environment | |  |  | |
| C10 | | Communicates effectively by telephone and electronic methods | |  |  | |
| C11 | | Demonstrates compassion, respect and a non judgemental approach towards patients and their informal carers | |  |  | |
| C12 | | Processes own reactions and remains respectful of patients and informal carers in stressful situations | |  |  | |
| Competencies: **Advance Care Planning and End of Life Tools** | | | | Pre Course | Post Course | |
| 1 – Not at all confident, 2 – A little confident, 3 – Quite confident, 4 – Very confident | | | | | | |
| ACP1 | Identifies when patients may want to discussion advance care planning to include preferred place care and death, involving the appropriate professional. | |  | | |  |
| ACP2 | Uses documentation, whether paper or electronic, appropriately to access inform and record end of life care planning and delivery succinctly and legibly as per NMC Guidance (NMC 2008) | |  | | |  |
| ACP3 | Is involved in advance care planning as an on-going process, | |  | | |  |
| ACP4 | Can state their understanding of different elements of Advance care planning:  Advance Statement  Advance decision to refuse treatment (ADRT)  Best Interests  Lasting power of attorney. Finance and welfare  Informed consent. | |  | | |  |
| ACP5 | Identifies the principals of the Gold Standards Framework and works with colleagues and patients to deliver care in this framework. | |  | | |  |
| ACP6 | Contributes to the team’s assessment in recognising the dying phase and works with colleagues, patients and relatives to introduce the Liverpool Care Pathway. | |  | | |  |

Post Course Practice Example:

Please discuss and give an example of how your end of life care practice has changed since doing the programme
