# Supplementary material for: Blended e-learning and end of life care in nursing homes: a small-scale mixed-methods case study
Source: BMC Palliat Care. 2014 Jun 16;13:31. doi: 10.1186/1472-684X-13-31 (PMC4080686; doi:10.1186/1472-684X-13-31)
Supplement: Additional file 2 — E-learning in end of life care study pre and post course questionnaire. [file 1472-684X-13-31-S2.doc]

**Blended e-learning improve end of life care in nursing homes:**

**A small-scale mixed-methods case study**

**Additional file 2: E-Learning in End of Life Care Study Pre and Post Course Questionnaire**

The following items were utilized in the 8-item author-designed pre- and post-course freetext questionnaire administered to ABC course participants. In the original questionnaire, each item was followed by a freetext box. The content of each item was derived from reading of relevant literature in the end of life and palliative care fields.

- What is the appropriate time to initiate end of life care?
- What are the main aims of end of life care?
- What kinds of tasks does end of life care involve, and what tools and care pathways are appropriate?
- Whose responsibility is end of life care? Are some occupations and/or care settings particularly important?
- What national guidelines, policies and networks are in place surrounding end of life care?
- How necessary is it to have specialist training in end of life care?
- What sources of information are there regarding end of life care?
- What contribution can e-learning make to training in end of life care and other topics?
