# Supplementary material for: Blended e-learning and end of life care in nursing homes: a small-scale mixed-methods case study
Source: BMC Palliat Care. 2014 Jun 16;13:31. doi: 10.1186/1472-684X-13-31 (PMC4080686; doi:10.1186/1472-684X-13-31)
Supplement: Additional file 3 — Items in pre and post course deceased patient record audit. [file 1472-684X-13-31-S3.doc]

**Blended e-learning improve end of life care in nursing homes:**

**A small-scale mixed-methods case study**

**Additional file 3: Items in Pre and Post Course Deceased Patient Record Audit**

This appendix contains the 21-item pre- and post-course patient record audit administered by ABC course facilitators in care and nursing homes before and after administering courses.

| Date Education Programme Commenced: Date Education Programme Completed: | | | | | | | | | | | | | | |
| --- | --- | --- | --- | --- | --- | --- | --- | --- | --- | --- | --- | --- | --- | --- |
| Demographics: | | | | | | | | | | | | | | |
|  | How many patients/ residents in the care home today are identified as being palliative? | |  | | | | | | | | | | | |
|  | Patients gender | | No of Male: No of Female: | | | | | | | | | | | |
| Diagnosis: | | | | | | | | | | | | | | |
|  | Primary Diagnosis and DOB of 5 palliative care patients / residents as documented in notes | | Secondary Diagnosis | | | | | | | Length of Stay in Care home /on Ward / known to community team (weeks) | | | | |
| A |  | |  | | | | | | |  | | | | |
| B |  | |  | | | | | | |  | | | | |
| C |  | |  | | | | | | |  | | | | |
| D |  | |  | | | | | | |  | | | | |
| E |  | |  | | | | | | |  | | | | |
| Advance Care Planning: Record: Yes/No/No Record | | | | | | | | | | | | | | |
| 4 | Is there evidence in the notes that the palliative care needs of the patient / resident were discussed in a ward round/ care home / GSF or MDT meeting? | | | a | | b | | c | | | d | | e | |
| 5 | Is there evidence in the notes that the patient was placed on a palliative care register or similar? | | |  | |  | |  | | |  | |  | |
| 6 | Is there evidence in the notes that a holistic assessment of the patient / resident has taken place? | | |  | |  | |  | | |  | |  | |
| 7 | Is there evidence in the notes that an advance care planning discussion was offered to the patient / resident? | | |  | |  | |  | | |  | |  | |
| 8 | Is there evidence in the notes of an advance care planning discussion? | | |  | |  | |  | | |  | |  | |
| 9 | Is there evidence in the notes of a preferred place of care? | | |  | |  | |  | | |  | |  | |
| 10 | Is there evidence in the notes of a preferred place of death? | | |  | |  | |  | | |  | |  | |
| 11 | Is there evidence in the notes of a statement of preferences and wishes? | | |  | |  | |  | | |  | |  | |
| 12 | Is there evidence in the notes of an Advance Decision to Refuse Treatment in place? | | |  | |  | |  | | |  | |  | |
| 13 | Did the patient / resident die in their recorded preferred place of death? | | |  | |  | |  | | |  | |  | |
| 14 | If ‘no’ to the above question – what were the reasons for not achieving preferred place of death? | | |  | |  | |  | | |  | |  | |
| Anticipatory Planning: Record: Yes/No/No Record | | | | | | | | | | | | | | |
| 15 | | Is there evidence in the notes of a symptom control assessment tool for this patient / resident? | | | a | | b | | c | | | d | | e |
| 16 | | Is there evidence in the notes of anticipatory drugs prescribed for this patient / resident in the dying phase? | | |  | |  | |  | | |  | |  |
| 17 | | Is there evidence in the notes of a syringe driver prescribed for the patient / resident? | | |  | |  | |  | | |  | |  |
| 18 | | Is there evidence in the notes that a syringe driver was dispensed for this patient/resident? | | |  | |  | |  | | |  | |  |
| 19 | | Is there evidence in the notes of a signed Do Not Attempt Resuscitation for this patient / resident? | | |  | |  | |  | | |  | |  |
| 20 | | Is there evidence in the notes of discussion with family / next of kin re deterioration of patient / resident? | | |  | |  | |  | | |  | |  |
| 21 | | Is there evidence in the notes the patient / resident commenced on the Liverpool Care Pathway or similar? | | |  | |  | |  | | |  | |  |
| Communication and Coordination: Record: Yes/No/No Record | | | | | | | | | | | | | | |
| 22 | | Is there evidence in the notes bereavement support and follow up was offered to the patient / resident’s family or next of kin? | | |  | |  | |  | | |  | |  |
| 23 | | Is there evidence that other services were involved with this patient / resident? | | |  | |  | |  | | |  | |  |
| 24 | | Is there evidence in the notes that other services involved were informed of the death? | | |  | |  | |  | | |  | |  |
|  | | | | | | | | | | | | | | |
| Name of Person Completing this template | | | | | Date of Completion | | | | | | | | | |
| Please now return to Project Lead – Thank you | | | | | | | | | | | | | | |
