# Supplementary material for: Blended e-learning and end of life care in nursing homes: a small-scale mixed-methods case study
Source: BMC Palliat Care. 2014 Jun 16;13:31. doi: 10.1186/1472-684X-13-31 (PMC4080686; doi:10.1186/1472-684X-13-31)
Supplement: Additional file 4 — Topic guide for interviews. [file 1472-684X-13-31-S4.doc]

**Blended e-learning improve end of life care in nursing homes:**

**A small-scale mixed-methods case study**

**Additional file 4: Topic Guide for Interviews**

The following topics, derived from reading relevant literature in end of life care, made up the semi-structured interview guide utilized by the author to conduct interviews with ABC course participants.

- Defining end of life care: appropriate time to initiate; main aims; tasks; tools and care pathways; professional responsibilities; guidelines and policies
- End of life care training: need for specialist training; current levels of training in the nursing home; sources of information about end of life care
- ABC course: general experience of course; perceptions of blended e-learning model in general, and online modules/facilitated workshops in particular; internet access and use; perceptions of course content; extent to which new skills, attitudes, knowledge gained; limitations
- Enablers of and barriers to long-term change: inter-professional collaboration; organizational aspects (meetings, discussion, management); workload and burnout; commitment to end of life care; availability of further training and resources
